# Executive function and endocrinological responses to acute resistance exercise

**DOI:** 10.3389/fnbeh.2014.00262

**Published:** 2014-08-01

**Authors:** Chia-Liang Tsai, Chun-Hao Wang, Chien-Yu Pan, Fu-Chen Chen, Tsang-Hai Huang, Feng-Ying Chou

**Affiliations:** ^1^Institute of Physical Education, Health and Leisure Studies, National Cheng Kung UniversityTainan, Taiwan; ^2^Department of Physical Education, National Kaohsiung Normal UniversityKaohsiung, Taiwan; ^3^Department of Recreational Sport and Health Promotion, National Pingtung University of Science and TechnologyPingtung, Taiwan; ^4^Chi Mei Medical CenterTainan, Taiwan

**Keywords:** resistance exercise, cognition, electrophysiological, behavior, cortisol, GH, IGF-1

## Abstract

This study had the following two aims: First, to explore the effects of acute resistance exercise (RE, i.e., using exercise machines to contract and stretch muscles) on behavioral and electrophysiological performance when performing a cognitive task involving executive functioning in young male adults; Second, to investigate the potential biochemical mechanisms of such facilitative effects using two neurotrophic factors [i.e., growth hormone (GH) and insulin-like growth factor-1 (IGF-1)] and the cortisol levels elicited by such an exercise intervention mode with two different exercise intensities. Sixty young male adults were recruited and randomly assigned to a high-intensity (HI) exercise group, moderate-intensity (MI) exercise group, and non-exercise-intervention (NEI) group. Blood samples were taken, and the behavioral and electrophysiological indices were simultaneously measured when individuals performed a Go/No-Go task combined with the Erikson Flanker paradigm at baseline and after either an acute bout of 30 min of moderate- or high-intensity RE or a control period. The results showed that the acute RE could not only benefit the subjects' behavioral (i.e., RTs and accuracy) performance, as found in previous studies, but also increase the P3 amplitude. Although the serum GH and IGF-1 levels were significantly increased via moderate or high intensity RE in both the MI and HI groups, the increased serum levels of neurotrophic factors were significantly decreased about 20 min after exercise. In addition, such changes were not correlated with the changes in cognitive (i.e., behavioral and electrophysiological) performance. In contrast, the serum levels of cortisol in the HI and MI groups were significantly lower after acute RE, and the changes in cortisol levels were significantly associated with the changes in electrophysiological (i.e., P3 amplitude) performance. The findings suggest the beneficial effects of acute RE on executive functioning could be due to changes in arousal, possibly modulated by the serum cortisol levels.

## Introduction

Participation in physical activity has been demonstrated to be associated with changes in cognitive performance involving executive functioning (Etnier et al., 1997). Accordingly, there have been many studies that attempt to explore the changes in cognitive performance that occur after a bout of acute exercise. With regard to resistance exercise, a growing number of work has strongly supported the view that executive functioning performance is enhanced via chronic resistance exercise (Perrig-Chiello et al., 1998; Ozkaya et al., 2005; Cassilhas et al., 2007; Liu-Ambrose et al., 2010), but that such a facilitative effect, as measured by behavioral indices, can also be found via acute resistance exercise (Chang et al., 2012, 2014). However, while previous studies have implicated the physiological (e.g., arousal) or hormonal (e.g., neurotrophic factors) responses to acute exercise intervention as the basis of any improvements in behavioral performance following physical exercise (Magnie et al., 2000; Joyce et al., 2009; Lambourne and Tomporowski, 2010; Dietrich and Audiffren, 2011; McMorris et al., 2011; Pesce et al., 2011; Tsai et al., 2014), no research has yet been conducted to explore the potential mechanisms underlying this process using electrophysiological and biochemical markers.

Cognitive performance after a bout of acute exercise could be influenced by the intensity of exercise, which could be attributed to the secreted levels of biochemical markers (e.g., neurotrophins and cortisol) or the states of arousal (Kashihara et al., 2009). Biopsychological arousal theory proposes that physiological responses to exercise mediate changes in a number of aspects of psychological functioning (e.g., cognitive functioning), through its direct effects on energetic arousal (EA) (Oweis and Spinks, 2001). However, the level of EA after exercise is strongly associated with the degree of exercise intensity (Thayer, 1989), with the proposed positive effects of a moderate arousal level on cognitive performance being based on the inverse-U theory (Oweis and Spinks, 2001). As the amount of biochemical markers that are secreted are closely related to the level of physical activity, the effects of acute resistance exercise on cognitive (i.e., behavioral and electrophysiological) performance may be particularly relevant to an investigation of the various components of exercise, including intensity. Given the conceptual links between the secreted levels of circulating biomarkers [e.g., cortisol, growth hormone (GH) and insulin-like growth factor-1 (IGF-1)] and resistance exercise intensity (Schwarz et al., 1996; McGuigan et al., 2004; Irving et al., 2009; Wahl et al., 2010), examining whether different intensities (high vs. moderate) of acute exercise influence the exercise-cognition relationship would seem logical in any attempt to understand how acute resistance exercise may benefit cognitive performance.

Cortisol, a glucocorticoid hormone produced by the adrenal cortex, is a corticosteroid released in response to stress as the end-product of the hypothalamic-pituitary-adreno-cortical (HPA) system (Henckens et al., 2012). Increased cortisol levels are often related to stress, which could result in dysfunction of neuronal plasticity, neurogeneis, or remodeling of the hippocampus, since the steroids inhibit glucose transport in hippocampal neurons and glia (Sapolsky, 1993; Duman, 2002). Cortisol can lead to arousal, as its release can limit the synthesis of the adrenocorticotrophin hormone (ACTH) and corticotrophin releasing hormone (CRH), both of which modulate arousal (Lambourne and Tomporowski, 2010). Although exercise is also thought of as a stressor, an acute bout of aerobic as well as resistance exercise can increase the arousal status and neural activation, which further facilitate the central executive function related to the hippocampus and frontal lobe (Magnie et al., 2000; Lambourne and Tomporowski, 2010; Dietrich and Audiffren, 2011; Pesce et al., 2011; Chang et al., 2012, 2014). Given the conceptual links between cortisol and exercise, cortisol may be related to the effects of exercise on cognition (Henckens et al., 2012). Indeed, a previous study found that the beneficial effects of a single bout of acute exercise on cognitive performance could be attributed to acute decreases in cortisol levels (Heaney et al., 2013). However, although earlier work has demonstrated that cortisol can modulate cognitive performance involving executive functions (e.g., inhibitive control, attention, and memory) (Vedhara et al., 2000; Henckens et al., 2012), the effects of cortisol levels on cognition follow a U-shaped curve (Lupien and McEwen, 1997), with moderate levels being positively associated with executive functioning (Blair et al., 2005), while highly elevated cortisol levels have been shown to interfere with the cognitive functions that are largely dependent on prefrontal networks (e.g., inhibitory control, attention regulation, and memory retrieval) (Kopell et al., 1970; Lupien and McEwen, 1997; Lyons et al., 2000; Quesada et al., 2012) and hippocampal functioning (e.g., declarative memory) (Almela et al., 2012). This is because the detrimental effects on such executive functions relying on these brain areas could, to some extent, be due to a pronounced cortisol stress response (Almela et al., 2012; Quesada et al., 2012) or the inhibition of dopaminergic reward-seeking systems (Tops et al., 2004).

Additionally, although the improvement in cognitive functioning following physical exercise could be attributed to changes in the hormonal response, some exercise-sensitive biomarker secretions seem to be characterized by different physiological and metabolic demands. For example, aerobic exercise can effectively increase serum brain-derived neurotrophic factor (BDNF) concentrations, whereas resistance exercise can effectively change serum GH and IGF-1 concentrations (Neeper et al., 1995; Cassilhas et al., 2007; Seo et al., 2010; Gregory et al., 2013). Indeed, resting IGF-1 concentrations are increased after short-term resistance training (Borst et al., 2001), but decreased after short-term endurance training (Nemet et al., 2004). Additionally, a single bout of resistance exercise, but not aerobic exercise, is a physiological stimulus for acute increases in GH and IGF-1 levels (Gregory et al., 2013). Taken together, these findings indicate that these circulating responses of neurotrophic factors are specific to different exercise modes.

GH and IGF-1 are signaling peptides which can cross the blood-brain barrier and bind to receptors in the central nervous system (Sonntag et al., 2005). Given the importance of the GH/IGF-1 axis for growth of glial cells, myelination, and neurons (Sonntag et al., 2005), the capacity of resistance exercise to alter GH and IGF concentrations might have important implications for cognitive performance. Indeed, there is growing evidence for a significant association between the GH/IGF-1 axis and such performance. Previous studies have demonstrated that serum IGF-1 levels (Rollero et al., 1998; Aleman et al., 1999, 2001; Kalmijn et al., 2000; Dik et al., 2003), GH levels (Quik et al., 2012), and the IGF-1/GH ratio (Morley et al., 1997) are associated with behavioral performance (e.g., information processing speed, target detection and response speed, short-term memory, and visual/auditory learning), and acute resistance exercise can significantly increase serum GH and IGF-1 levels (Nicklas et al., 1995; Rubin et al., 2005). We thus hypothesize that the changes in serum levels of GH and IGF-1 following a bout of acute resistance exercise would be positively correlated with the cognitive performance of executive functioning.

Previous studies have reported that executive functions are more strongly affected by physical activity or exercise than other aspects of cognitive functioning (Etnier et al., 1997; Colcombe and Kramer, 2003), and acute exercise has been suggested to selectively augment executive function performance involving inhibitory control and attention (Drollette et al., 2012). Previous studies have demonstrated that, relative to the resting session, young adults exhibited not only higher response accuracy and shorter reaction time (RT), but also larger P3 amplitudes (i.e., devoting more attentional resources) following a bout of acute aerobic exercise when performing a modified flanker task (Hillman et al., 2003; Kamijo et al., 2009). While P3 event-related potential is known to be related to inhibition and attentional resource allocation (Jonkman et al., 2003; Tsai et al., 2009), a better understanding of the electrophysiological changes (e.g., P3 amplitude) underlying any improvements in behavioral performance may provide insights into the specific component processes involved in cognitive control that are modulated by acute resistance exercise (Hillman et al., 2009).

Although a number of studies have demonstrated that a bout of acute resistance exercise can effectively enhance behavioral indices (Chang et al., 2012, 2014), thus far, no research has yet been conducted on the effects of such an acute exercise mode on electrophysiological performance. Therefore, the first aim of this study was to elucidate the effects of a bout of moderate- or high-intensity resistance exercise on behavioral (i.e., RT and accuracy) and electrophysiological (i.e., P3 amplitude) performance using a Go/No-Go task combined with the Erikson Flanker paradigm in young males. Since such a cognitive task involves the cognitive processes (i.e., inhibitory control and attention), and previous studies have demonstrated that the cognitive performance can effectively be enhanced in adolescents (Hogan et al., 2013) and young adults (Ruchsow et al., 2005) after a bout of acute exercise, we thus hypothesized that moderate- or high-intensity acute resistance exercise could produce different degrees of beneficial effects on cognition with regard to behavioral and electrophysiological performance in both exercise-intervention (EI) [i.e., moderate-intensity (MI) and high-intensity (HI)] groups relative to those seen in the non-exercise-intervention (NEI) group.

In addition, since no studies have examined the potential biochemical mechanisms underlying the beneficial effects of acute resistance exercise on cognitive performance, the second aim of this study was to explore further the issue. Based on previous research, we postulated that both EI groups would see different changes in serum levels of biochemical markers which would result in different effects on cognitive performance.

## Materials and methods

### Participants

Sixty male participants aged between 20 and 29 were recruited from the same university and randomly assigned to a high-intensity (HI) exercise group (*n* = 20), moderate-intensity (MI) exercise group (*n* = 20), and non-exercise-intervention (NEI) group (*n* = 20). Only male participants were selected in the current study, because research has shown that gender differences exist in the responses to resistance training, such as those that affect endocrine functioning (Staron et al., 1994). Thus a mixed-gender group may lead to disproportionate improvements in muscle and metabolic functions between male and female participants, which would presumably affect any related cognitive changes with regard to both cognitive and biochemical indices (Rubia et al., 2010). All participants were non-smokers, right-handed, as assessed by a handedness inventory (Chapman and Chapman, 1987), and had normal or corrected-to-normal vision. They were asked to complete a medical history and demographic questionnaire, and reported being free of any psychiatric or neurological disorders, cardiovascular or metabolic diseases, or medication intake that would influence central nervous system functioning. Additionally, they completed the International Physical Activity Questionnaire (IPAQ) (Craig et al., 2003) and the Physical Activity Readiness Questionnaire (PARQ) (Thomas et al., 1992) to avoid potential risk factors that might be exacerbated during acute resistance exercise. None of the participants showed any symptoms of cognitive impairment or depression, as separately measured by the Mini-Mental State Examination (MMSE, all scored above 24) (Folstein et al., 1975) and Beck Depression Inventory II (DBI-II, all scored below 13) (Beck et al., 1996). All the participants provided written informed consent to participate in the experiment, which was approved by the Institutional Ethics Committee. As shown in Table 1, the three groups were matched in age, body mass index (BMI), BDI-II, MMSE, and IPAQ, as well as resting HR (all *p* > 0.05).

**Table 1:** **Demographic characteristics (mean ± *SD*) of the two exercise intervention groups and one non-exercise-intervention group**.

**Group**	**MI (*n* = 20)**	**HI (*n* = 20)**	**NEI (*n* = 20)**
Age (years)	23.15 ± 2.52	22.40 ± 2.44	23.20 ± 2.12
Body mass index (kg/m^2^)	20.83 ± 1.45	21.53 ± 1.80	22.03 ± 2.57
Education (years)	16.50 ± 1.67	17.10 ± 1.83	17.35 ± 1.95
MMSE	29.10 ± 0.91	29.25 ± 0.97	28.90 ± 0.91
BDI-II	4.80 ± 2.80	4.35 ± 2.50	4.05 ± 2.86
Resting HR (beats/min)	66.00 ± 5.18	67.75 ± 9.39	66.65 ± 6.21
IPAQ (MET-min/week)	1091.58 ± 459.51	888.20 ± 292.65	933.23 ± 240.95

HI, high-intensity; MI, moderate-intensity; NEI, non-exercise-intervention; MMSE, Mini Mental State Examination; BDI, Beck Depression Inventory; HR: Heart Rate; IPAQ: International Physical Activity Questionnaire. Three groups were not significantly different at baseline for all variables.

### Procedure

The participants were required to make two visits to the cognitive neurophysiology laboratory. On the first visit the research assistant explained the experimental procedure, and asked the participants to complete an informed consent form, a medical history and demographic questionnaire, and MMSE, DBI-II, IPAQ, and PARQ. Their height and weight were also measured to calculate their BMI. Two certified fitness instructors then completed all assessments of one-repetition maximum (1-RM) and peak muscle power for each participant. All participants in the MI and HI groups were familiarized with the exercise machines before the acute exercise intervention took place.

The second visit took place in the morning 2 days later, and to prepare for this the participants were asked to refrain from strenuous exercise and alcohol intake for 24 h, and food and caffeine were also prohibited for 3 h before exercising, since both caffeine and food consumption are associated with increases in P3 amplitude (Geisler and Polich, 1990; Dixit et al., 2006) and biochemical makers (e.g., cortisol) (Wu, 2014). When arriving at the laboratory, each participant was fitted with a Polar heart rate (HR) monitor (RX800CX, Finland), and was then asked to sit in an adjustable chair in front of a computer screen (with a width of 43 cm) in an acoustically shielded room with dimmed lights. Body temperature and resting HR were measured. An electrocap and electro-oculographic (EOG) electrodes were attached to each participants' scalp and face before the cognitive task test. The viewing distance was approximately 75 cm. Ten practice trials were carried out to familiarize the participants with the procedure of the cognitive task. Blood was then withdrawn and the formal cognitive test was immediately administered and electrophysiological signals recorded. The HI and MI groups then performed approximately 40 min of high-intensity (80% 1RM) and moderate-intensity (50% 1RM) acute resistance exercise on the exercise machines, respectively, with 10 min of warm-up and 30 min of core content. The core resistance exercise consisted of the following exercises in the order stated: bench presses, biceps curls, triceps extensions, leg presses, vertical butterflies, and leg extensions. Both HI and MI groups performed the resistance exercise for two sets of 10 repetitions, at an average speed, with a 90-s rest between sets, and a 2-min interval between each different exercise. Since exercise-induced hyperthermia and tachycardia are associated with systematic changes in P3 component (e.g., decrease in P3 latency) (Geisler and Polich, 1990), body temperature was measured and HR was assessed with a Polar HR monitor after the acute resistance exercise, with both measurements taken every 3 min. Once the participants' body temperature and HR had returned to within 10% of pre-exercise levels (on average about 5 min after a bout of acute resistance exercise), blood was immediately withdrawn from them, and they then completed the cognitive task as their event-related potentials (ERPs) were recorded. Additional blood samples were then taken after the subjects had completed the cognitive task.

With regard to the NEI group, after the first cognitive test they took a rest of about 45 min, during which they read magazines, and they completed the cognitive test again. All of the participants performed the cognitive test at the same time of day to control for circadian distortions.

### Cognitive task paradigm

Since acute exercise has been suggested to selectively augment executive functioning performance involving inhibitory control and attention (Drollette et al., 2012), a Go/No-Go task combined with the Erikson Flanker paradigm was used in this study (Ruchsow et al., 2005). Eight different letter strings (i.e., congruent: UUUUU, BBBBB, VVVVV, DDDDD; and incongruent: VVUVV, DDBDD, DDVDD, BBDBB) were presented on the computer screen in a randomized order with equal probability. Participants had to focus on the target letter in the middle of an array. Upon the appearance of letters U and B (Go condition), the participants had to respond as quickly and accurately as they could, using their right index finger to press the “M” button of the keyboard and their left index finger to press the “X,” respectively. In contrast, they were told not to press any key if the letters D and V appeared in the middle of an array (No-Go condition). The whole experiment consisted of two blocks with 200 trials each, with 200 Go trials and 200 No-Go trials. All letter strings were presented in white text and for 200 ms against a black background on a laptop computer monitor. The participants had to respond within 1800 ms. There was an interval of 2000 ms between each trial. All participants performed the cognitive task with concomitant electrophysiological recording. After a practice block of 10 trials to make sure the participants understood the whole experimental procedure, the formal test was administered to collect RTs, accuracy rate, and ERPs data. The total duration of the cognitive test was approximately 15 min.

### Electrophysiological recording and analysis

Electroencephalographic (EEG) activity was recorded using 18 electrode sites (F7, F8, F3, F4, Fz, T3, T4, C3, C4, Cz, T5, T6, P3, P4, Pz, O1, O2, Oz) mounted in an elastic electrode cap (Quik-Cap, Compumedics Neuroscan, Inc., El Paso, TX) designed for the International 10-20 System. To monitor possible artifacts due to eye movements, horizontal and vertical bipolar electrooculographic activity (HEOG and VEOG) was recorded using adhesive electrodes placed on the supero-lateral right canthus and below and lateral to the left eye. Scalp locations were referred to linked mastoid electrodes, while a ground electrode was placed on the mid-forehead on the Quik-Cap. All electrode impedances were below 5 kΩ. EEG data acquisition employed an A/D rate of 500 Hz/channel, a band-pass filter of 0.1–50 Hz, and a 60 Hz notch filter, with continuous writing to hard disk for off-line analysis using SCAN4.3 analysis software (Compumedics Neuroscan, Inc., El Paso, TX) (Tsai et al., 2014).

Trials with a response error or RT quicker than 150 ms or slower than 800 ms, which were regarded as anticipatory or delay errors, respectively were excluded from the analysis. This time window was able to exclude all responses greater than two standard deviations from the mean response of each group, thereby excluding outliers that could skew the group means. The ERP epoch was defined as 200 ms pre-stimulus to 1200 ms post-stimulus onset. During the recording epoch, trials containing ocular artifacts were also discarded from further analysis, with a threshold of 100 μ V in the vertical and horizontal electrooculograms being set for this. The remaining effective ERPs data was assembled across epochs according to different conditions. The stimulus-elicited P3 component, defined as the major positive deflection after the stimulus over the scalp (i.e., Fz, Cz, and Pz) occurring 250–500 ms for Go-P3 and 350–550 ms for No-Go-P3, was distinguished and averaged across the three electrodes, with correction for differences in the 200 ms pre-stimulus baseline (Kato et al., 2009).

### Blood sampling and analysis

Blood samples were obtained at three time points (T1: before the 1st cognitive task test; T2: before the 2nd cognitive task about 5 min after acute exercise; and T3: immediately after the 2nd cognitive task test) by a phlebotomist. The blood samples were withdrawn from the antecubital vein via an aseptic technique for analysis of serum IGF-1, GH, and cortisol. During the T2 and T3 time points, blood samples were obtained via an indwelling catheter located in a forearm vein. Following each sample collection, the catheter was flushed with sterile saline to prevent clot formation, and the catheter was cleared of saline prior to each sample collection. The blood was allowed to clot (BD Vacutainer Plus), and then centrifuged at 3000 rpm for 15 min at 4°C (Hettich Mikro 22R, C1110). Each sample was frozen and stored at −80°C for further serum marker assays. Serum values of GH and IGF-I were determined by a chemiluminescence immunoassay method using an Access Ultrasensitive hGH reagent pack (Beckman Coulter Inc, USA) and Liaison IGF-1 reagent (DiaSorin S.P.A., Italy), respectively. The levels of serum cortisol were analyzed by an enzyme-linked immunosorbent assay (ELISA) using cortisol kits (JL840685/R06, Abbott, Abbott Park, Illinois, USA). The detection limit for GH by this method was 0.002 ng/mL for GH and 3 ng/mL for IGF-1. The whole procedure for the determination of the three biochemical markers was performed by the same person to avoid inter-operator bias.

### Statistical analysis

For the behavioral (i.e., RTs and accuracy) and electrophysiological (i.e., P3 amplitude) analysis, all independent variables from the acute bout of resistance exercise were analyzed with a three-way repeated measures analysis of variance (ANOVA) [i.e., time (pre- vs. post-exercise) × group (HI vs. MI vs. NEI) × conditions (behavioral: congruent-go vs. incongruent-go; electrophysiological: congruent-go vs. congruent-no-go vs. incongruent-go vs. incongruent-no-go)]. Where a significant difference occurred, Bonferroni *post-hoc* analyses were performed. For the serum analysis, two-way repeated ANOVA with *post-hoc* Bonferroni were used to assess both the effects of time (T1 vs. T2 vs. T3) and group (MI vs. HI vs. NEI). Homogeneity and normality of variance assumptions were confirmed by Levene's and Kolmogorov-Smirnov tests, respectively. The significance levels of the *F* ratios were adjusted with the Greenhouse-Geisser correction for the violation of the assumption of sphericity when the degrees of freedom were more than one. The effect size (i.e., partial η^2^: η^2^_*p*_) is also reported to complement the use of significance testing. The following conventions were adopted to determine the magnitude of the mean effect size: <0.08 (small effect size), between 0.08 and 0.14 (medium effect size), and >0.14 (large effect size). A value of *p* < 0.05 was considered to be significant.

## Results

### Behavioral performance

#### Reaction time

**Figure 1:**
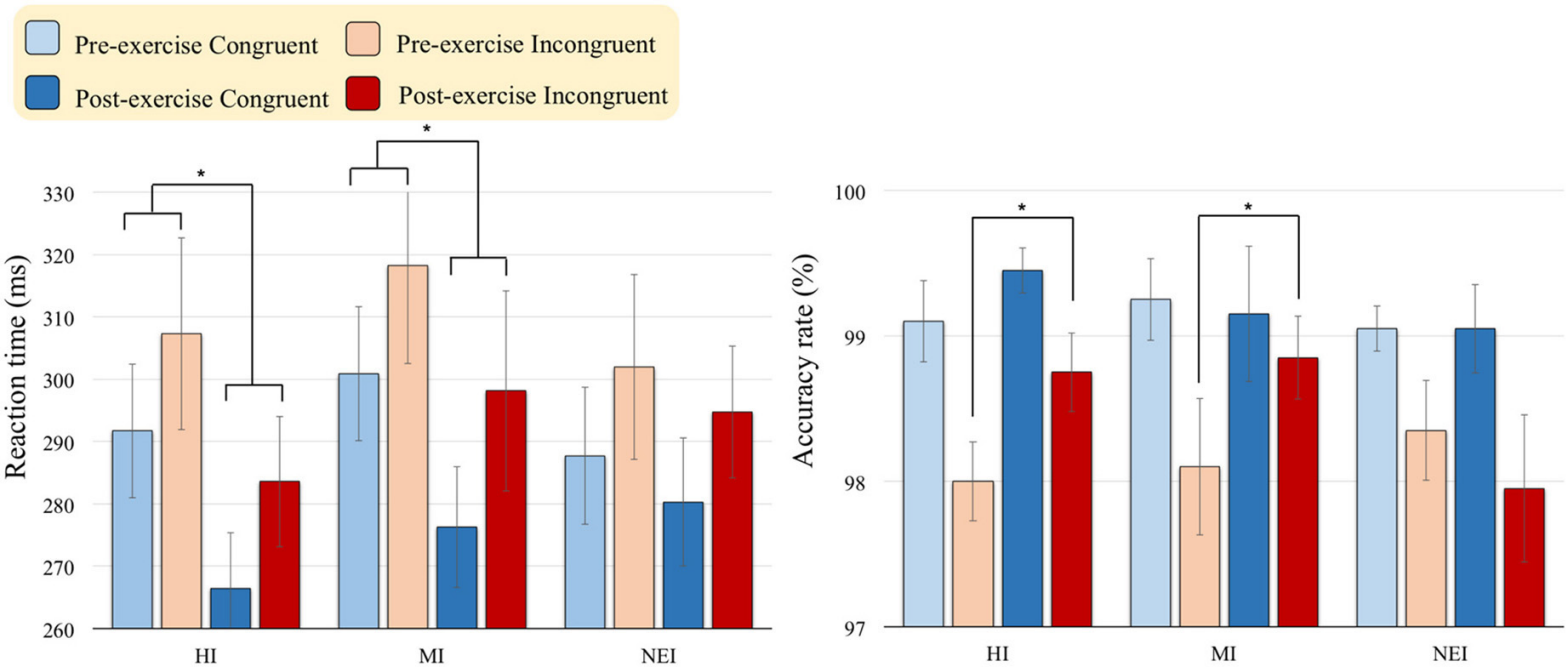
Behavioral [RTs (ms) in Go conditions; Accuracy rate (%) in No-Go conditions] performance (Mean ± *SD*) for the two exercise intervention groups (i.e., HI: high-intensity and MI: moderate-intensity) before and after an acute bout of resistance exercise and one non-exercise-intervention (NEI) group before and after rest (^*^ < 0.05).

As shown in Figure 1, there were main effects of *Time* [*F*_(1, 57)_ = 42.25, *p* < 0.001, η^2^_*p*_ = 0.43] and *Condition* [*F*_(1, 57)_ = 221.94, *p* < 0.001, η^2^_*p*_ = 0.80] on RTs, suggesting that RTs were faster after (283.24 ms) than before (301.29 ms) acute exercise, and that RTs were faster in the congruent-go (283.89 ms) than in the incongruent-go (300.65 ms) conditions. The effects of the interaction of *Time* × *Group* [*F*_(2, 57)_ = 3.78, *p* = 0.029, η^2^_*p*_ = 0.12] on RTs was also significant. *Post-hoc* analyses showed RTs were significantly faster after compared to before acute exercise in both HI (pre- vs. post-exercise = 299.49 ± 47.90 vs. 275.02 ± 47.56 ms, *p* < 0.001) and MI (pre- vs. post-exercise = 309.57 ± 69.15 vs. 287.21 ± 68.91 ms, *p* < 0.001) groups in the Go condition.

#### Accuracy rate

There was a main effect of *Condition* [*F*_(1, 57)_ = 23.11, *p* < 0.001, η^2^_*p*_ = 0.29] on the accuracy rate, suggesting that the accuracy rate of the No-Go condition was higher in the congruent condition (99.2%) than in the incongruent one (98.3%). The effects of the interactions of *Time* × *Group* [*F*_(2, 57)_ = 3.61, *p* = 0.033, η^2^_*p*_ = 0.11] and *Time* × *Group* × *Condition* [*F*_(2, 57)_ = 3.22, *p* = 0.048, η^2^_*p*_ = 0.10] on the accuracy rate were also significant. *Post-hoc* analyses showed the accuracy rate in the incongruent-no-go condition was significantly higher after compared to before acute exercise in both HI (pre- vs. post-exercise = 98.00 ± 2.03% vs. 98.75 ± 1.21%, *p* = 0.024) and MI (pre- vs. post-exercise = 98.10 ± 2.10% vs. 98.85 ± 1.27%, *p* = 0.015) groups.

### Electrophysiological performance

**Figure 2:**
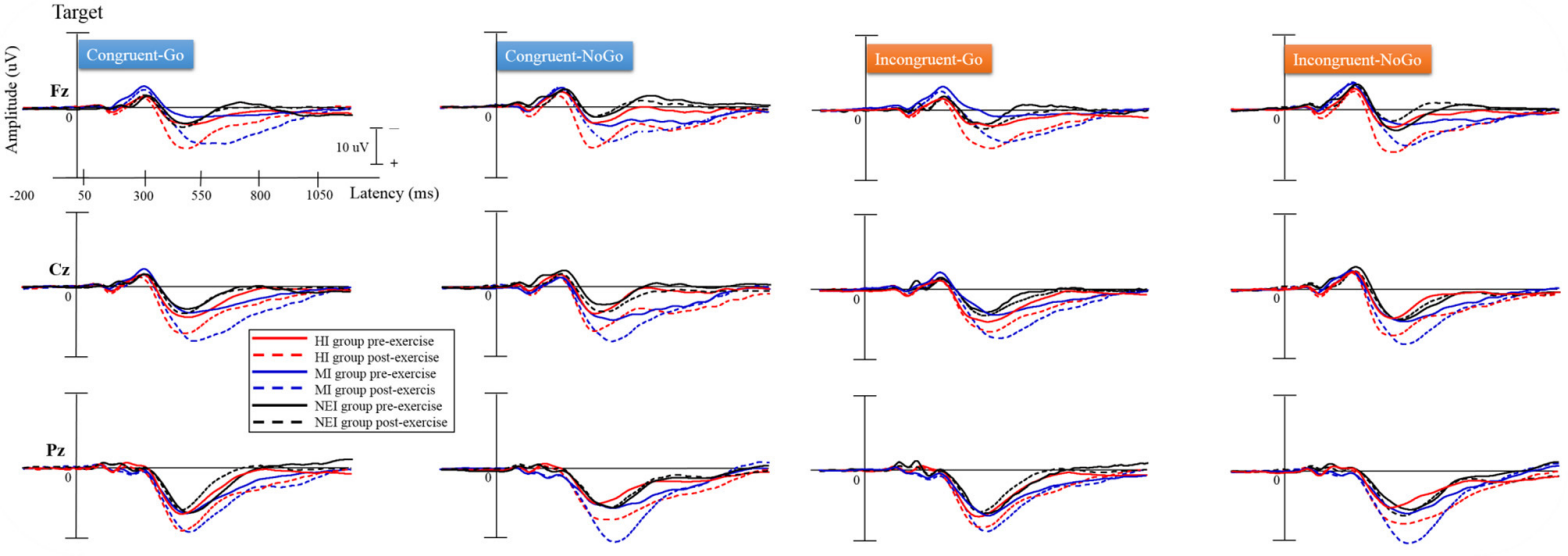
**Grand averaged ERP waveforms (Fz, Cz, and Pz) in the congruent-go, congruent-no-go, incongruent-go, and incongruent-no-go conditions for the two exercise intervention (i.e., HI: high-intensity and MI: moderate-intensity) groups before and after an acute bout of resistance exercise and one non-exercise-intervention (NEI) group before and after rest**.

The grand averaged ERP waveforms obtained for the three groups are shown in Figure 2. There were significant effects of *Group* [*F*_(2, 57)_ = 3.34, *p* = 0.042, η^2^_*p*_ = 0.11] and *Time* [*F*_(2, 57)_ = 39.91, *p* < 0.001, η^2^_*p*_ = 0.41] on P3 amplitudes, suggesting that no significant differences were observed between the three groups in the averaged P3 amplitude across the four conditions before acute exercise. In addition, the HI (13.58 ± 7.35 μ V) and MI (14.91 ± 4.49 μ V) groups showed significantly larger P3 amplitudes than the NEI (8.34 ± 3.42μ V) group (HI vs. NEI, *p* = 0.012; MI vs. NEI, *p* = 0.001). The effect of the interaction of *Group* × *Time* [*F*_(2, 57)_ = 7.10, *p* = 0.002, η^2^_*p*_ = 0.20] on P3 amplitudes was also significant. *Post-hoc* analyses revealed that the P3 amplitudes were significantly larger after compared to before acute exercise in both HI (pre- vs. post-exercise: 8.24 ± 6.98 vs. 13.58 ± 7.35 μ V, *p* < 0.001) and MI (pre- vs. post-exercise: 8.40 ± 4.86 vs. 14.91 ± 4.49 μ V, *p* < 0.001) groups.

### Biochemical indices

#### Growth Hormone (GH)

**Figure 3:**
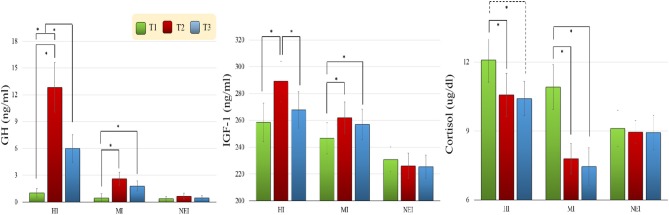
Changes in growth hormone (GH), insulin-like growth factor-1 (IGF-1), and cortisol levels (Mean ± *SE*) for the two exercise intervention (i.e., HI: high-intensity and MI: moderate-intensity) groups before [T1 (green bar)] and after [T2 (red bar): before the 2nd cognitive task; T3 (blue bar): after the 2nd cognitive task] an acute bout of resistance exercise and one non-exercise-intervention (NEI) group before and after rest (^*^p < 0.05).

As seen in Figure 3, there were significant effects of *Group* [*F*_(2, 57)_ = 13.56, *p* < 0.001, η^2^_*p*_ = 0.32] and *Time* [*F*_(2, 114)_ = 22.53, *p* < 0.001, η^2^_*p*_ = 0.28], and a significant effect of *Group* × *Time* [*F*_(4, 114)_ = 12.83, *p* < 0.001, η^2^_*p*_ = 0.31], on GH levels. *Post-hoc* analyses revealed that no significant differences in the GH levels were observed between three groups at the T1 time point. The GH levels at the T2 (HI vs. MI: *p* < 0.001; HI vs. NEI: *p* < 0.001) and T3 (HI vs. MI: *p* = 0.013; HI vs. NEI: *p* = 0.001) time points were found to be significantly higher in the HI group as compared to the NEI and MI groups. In addition, in the HI group, the GH level was found to increase significantly at both the T2 and T3 time points relative to the T1 time point (T1 vs. T2: *p* < 0.001; T1 vs. T3: *p* = 0.004), and decrease significantly at the T3 relative to the T2 time point (*p* = 0.003). Moreover, in the MI group, the GH level was found to increase significantly at both the T2 and T3 time points relative to the T1 time point (T1 vs. T2: *p* = 0.003; T1 vs. T3: *p* = 0.020). No significant correlations emerged among the changes in GH levels and changes in behavioral and electrophysiological performances with acute exercise in any of the EI groups.

#### Insulin-like growth factor-1 (IGF-1)

There were significant effects of *Group* [*F*_(2,57)_ = 3.83, *p* = 0.028, *η _*p*_*^2^ = 0.12] and *Time* [*F*_(2,114)_ = 10.14, *p* < 0.001, *η _*p*_*^2^ = 0.15] on IGF-1 levels, as well as a significant *Group* × *Time* effect [*F*_(4,114)_ = 5.90, *p* < 0.001, *η _*p*_*^2^ = 0.17]. *Post-hoc* analyses revealed that no significant differences were found for the T1 time point among the three groups. However, the IGF-1 levels at the T2 (*p* = 0.003) and T3 (*p* = 0.039) time points were found to be significantly higher in the HI group as compared to the NEI group. In addition, in the HI group, the serum IGF-1 level was found to increase significantly at the T2 time point relative to T1 (*p* < 0.001), and to decrease significantly at T3 relative to T2 time point (*p* = 0.002). Moreover, in the MI group the serum IGF-1 level was found to increase significantly at the T2 and T3 time points relative to the T1 time point (T1 vs. T2: *p* = 0.010; T1 vs. T3: *p* = 0.005). No significant correlations emerged among the changes in IGF-1 levels and changes in behavioral and electrophysiological performances with acute exercise in any of the EI groups.

#### Cortisol

There was a significant effect of *Time* [*F*_(1,57)_ = 21.40, *p* < 0.001, η ^2^_*p*_ = 0.27] on cortisol levels, and a significant *Group* × *Time* effect [*F*_(4,114)_ = 6.10, *p* = 0.001, *η _*p*_*^2^ = 0.18]. *Post hoc* analyses revealed that no significant differences were observed between the cortisol levels of the three groups at the T1 time point. However, in the HI group the cortisol level was found to decrease significantly at the T2 time point (T1 vs. T2: *p* = 0.023) and approach significance at the T3 time point (T1 vs. T3: *p* = 0.062) relative to T1. In the MI group the cortisol level was found to decrease significantly at both the T2 and T3 time points relative to T1 (both T1 vs. T2 and T1 vs. T3: *p* < 0.001). The serum cortisol levels between T2 and T3 did not change significantly.

Additionally, the correlations achieved significance with regard to the changes in cortisol levels and electrophysiological performance (i.e., P3 amplitude) with acute exercise in the MI (T2 vs. T1: *r* = −0.50, *p* = −024; T3 vs. T1: *r* = −0.51, *p* = 0.020) and HI (T2 vs. T1: *r* = −0.49, *p* = 0.029; T3 vs. T1: *r* = −0.58, *p* = 0.007) groups.

## Discussion

The purposes of the present study were to investigate the effects of acute resistance exercise on executive functions when individuals performed a Go/No-Go task combined with the Erikson Flanker paradigm, and to explore the potential biochemical mechanisms in relation to two neurotrophic factors (i.e., GH and IGF-1) and the cortisol biomarker. Even though the young males performed almost at ceiling and showed fast responses before the intervention when performing the cognitive task, acute resistance exercise, irrespective of high or moderate exercise intensity, could still affect cognitive [behavioral (e.g., RTs and accuracy rate) and electrophysiological (i.e., P3 amplitude)] performance when they performed an executive function/attentional control task. In terms of biochemical markers, such exercise prescriptions significantly increased the serum levels of two neurotrophic factors (i.e., GH and IGF-1) and lower the serum levels of cortisol in both EI groups compared to baseline. Only the cortisol levels remained stable for about 20 min after exercise, and the correlations between changes in serum cortisol levels and changes in electrophysiological (i.e., P3 amplitude) performance reached a significant level in the both EI groups.

Several experimental studies have demonstrated that acute aerobic exercise can improve cognitive performance when the subjects performed cognitive tasks involving executive functioning (Hillman et al., 2003, 2009; Davranche et al., 2009; Tsai et al., 2014). Recently, two studies also demonstrated that acute resistance exercise could significantly cause shorter RTs and higher accuracy rates when the participants performed the executive functioning tasks (e.g., the Stroop test and the Tower of London task), suggesting that such an exercise intervention could improve behavioral performance (Chang et al., 2012, 2014). In line with the findings of these previous studies, the RTs in the present study were significantly improved in both EI groups when performing the modified Flanker task after acute resistance exercise compared to pre-exercise. In addition, the finding that the accuracy rate in the incongruent-no-go condition was significantly higher after compared to before acute resistance exercise in both EI groups was also in agreement with that of a previous work (Hillman et al., 2009) which found that response accuracy was generally improved following acute aerobic exercise compared to pre-exercise, with better performance during incongruent conditions requiring greater amounts of inhibition when performing a modified Flanker task. Indeed, previous studies also reported that the beneficial effects of acute exercise could be confined to inhibitory control and attention (e.g., Drollette et al., 2012). However, given the results of the present study, where exercise had similar effects on behavioral performance in both EI groups, it appears that, regardless of whether moderate or high intensity, acute resistance exercise could be a viable approach to enhancing the executive functions involving inhibitory control and attention in young male adults. The improved behavioral performance with regard to central executive functioning found in this work could be related to increases in neural activation or general physiological arousal (Magnie et al., 2000; Joyce et al., 2009; Lambourne and Tomporowski, 2010; Dietrich and Audiffren, 2011; Pesce et al., 2011).

Indeed, the amplitude of the P3 potential increased following a bout of acute resistance exercise relative to the pre-exercise levels in both the EI groups in the present study, in a similar manner to the beneficial effects of acute aerobic exercise reported in previous studies (Hillman et al., 2003, 2009; Tsai et al., 2014). Since the P3 amplitude is proportional to the amount of attentional resources allocated to a task (Tsai et al., 2009), the findings of the current study suggest that young male adults could attain more efficient cognitive processing when performing the cognitive task after a bout of acute resistance exercise. In addition, this study also found that significant increases in P3 amplitude were observed across all three cortical sites (Fz, Cz, and Pz), supporting Polich and Kok's (1995) view that, since variations in scalp topography were not observed following fluctuations in biological state in their work, the effects of P3 amplitude and exercise occur in a global fashion. Moreover, Yanagisawa et al. (2010) used multichannel functional near-infrared spectroscopy (fNIRS) measurements and found that acute exercise could improve executive functions and increase cortical activation, suggesting that significant increases in oxy-Hb signaling could be the potential mechanism underlying such changes. Therefore, the increased oxygenation and flood flow in the brain after acute exercise is likely the cause of the neural activation (i.e., greater P3 amplitudes) found in the present study. Additionally, recent research found that glucose moderates the magnitude of the P3 ERP component when individuals performed the oddball task (Riby et al., 2008) and acute resistance exercise can facilitate blood glucose control and insulin secretion (Balaguera-Cortes et al., 2011; Moreira et al., 2012). It is possible that, since performing the executive function task in the present study requires simultaneous inhibitory control and attention, greater demands on metabolic resources could be compensated following a single bout of resistance exercise. Such an effect further facilitated the P3 performance. Another potential mechanism for the facilitative effects of acute resistance exercise on electrophysiological performance and executive function performance may be the changes in biochemical markers (i.e., GH and IGF-1) that occur in the central nervous system (Kashihara et al., 2009).

In this study we investigated two neurotrophic factors (i.e., GH and IGF-1) which play central roles in the health of neurons in the brain, since previous studies investigating the effect of resistance training on executive functions mostly discussed the potential mechanisms using these (Cassilhas et al., 2007, 2010; Seo et al., 2010). This is perhaps due to the fact that the secretions of these two biomarkers are exercise-sensitive, and occur in relation to specific physiological and metabolic demands (Gregory et al., 2013). In the current study, serum GH and IGF-1 levels were significantly increased in both EI groups after acute resistance exercise, supporting the findings of previous studies which demonstrated that a single bout of resistance training can significantly increase serum GH levels, which could produce a subsequent increase in its secondary mediator (i.e., IGF-1) (Nicklas et al., 1995), and that trained men could increase circulating IGF-1 responses with a bout of acute resistance exercise (Rubin et al., 2005). However, this study found that changes in the levels of both neurotrophic factors were not significantly correlated with the changes in behavioral and electrophysiological performance in the healthy young male adults when performing the cognitive task. These results do not stand alone, and are somewhat in agreement with prior studies which found that there was no relationship between serum IGF-1/GH concentrations and specific aspects of cognitive-behavioral measurements and electrophysiological performance (e.g., N2b) when middle-aged to elderly adults performed a Go/No-Go task (Papadakis et al., 1995; Aleman et al., 1999; Quik et al., 2012). The possible explanations for the lack of correlation are as follows: (1) the serum levels of GH and IGF-1 were significantly decreased from the T2 to the T3 time points in this study; (2) even though the serum GH and IGF-1 levels increased much more after high-intensity resistance exercise in the HI group compared to the MI one, these changes still did not show a very strong positive correlation with the changes in cognitive performance; and (3) the beneficial effects of acute resistance exercise on behavioral and electrophysiological performance might be explained in terms of heightened arousal, due to exercise-induced changes in reallocation of mental resources and metabolic rate (Audiffren, 2009). However, it is worth pointing out that GH and IGF-1 are important molecular mediators of neural efficiency in the human brain (Sonntag et al., 2005). Previous studies demonstrated that regular, long-term resistance exercise is associated with increases in circulating GH and IGF-1 concentrations in young males (Ballard et al., 2005; Willoughby et al., 2007), and that changes in the resting serum IGF-1 concentrations after 12 months of resistance exercise were significantly correlated with the changes in RTs and P3 amplitude in healthy elderly subjects (Tsai et al., under review). We can thus not negate the potential roles of GH and IGF-1 in the beneficial effects of regular, long-term resistance exercise on cognitive functioning, since these acute effects in the present study could be washed out due to the limited timeframe of exercise.

Given that cortisol is indicative of arousal, the results of this study support the view that the participants' level of arousal was altered after the acute exercise intervention, and this seems to have positively affected their cognitive processes (Lambourne and Tomporowski, 2010), leading to faster RTs and a higher accuracy rate following a bout of acute resistance exercise in both MI and HI groups. Since high levels of cortisol have a detrimental effect on executive functions (e.g., inhibitory control and attention regulation) (Kopell et al., 1970; Lupien and McEwen, 1997; Lyons et al., 2000), the beneficial effects of acute resistance exercise on behavioral and electrophysiological performance for both MI and HI groups in the present study could be attributed to the reduced cortisol levels. The post-exercise concentrations of cortisol that were found in the present study were significantly lower than the pre-exercise ones in both EI groups, echoing earlier studies which found that the cortisol concentration is modulated by acute exercise, and significantly decreases immediately post-exercise and for up to 1–2 h post-exercise compared to pre-exercise (Kemmler et al., 2003; Heaney et al., 2013). Henckens et al. (2012) found that cortisol can modulate emotional and attentive processing, and that high circulating corticosteroid levels could negatively influence the function of the amygdala in executive networks. In addition, lower circulating corticosteroid levels modulate the neural correlates of sustained attention by reducing cuneus activity, which might shift the brain back from a stimulus-driven response mode to a more controlled mode, and restore proper brain functioning in the aftermath of stress. It is worth pointing out that the serum cortisol level was found to significantly decrease at T2 and T3 relative to the T1 time point, and did not significantly change between T2 and T3 in either EI group in the present study, suggesting that the serum cortisol levels had stabilized by at least 20 min after resistance exercise. These findings might partly support the relationship between changes in cortisol levels and cognitive performance.

However, while the changes in cortisol levels were significantly correlated with those in P3 amplitude, they were not significantly correlated with the changes in RTs and accuracy rate in either the MI and HI groups in the current study, suggesting such a biochemical marker might be more sensitive to the electrophysiological index relative to the behavioral measures. Indeed, cortisol is associated with an increased activation of the alerting/arousal component of attention (Lambourne and Tomporowski, 2010; Schulz et al., 2013), and thus resting EEG has been shown to be related to cortisol levels (Schulz et al., 2013). Previous studies also found that exercise may serve to increase neuronal synchrony, as alpha wave activation increased after a bout of acute exercise (Kubitz and Mott, 1996; Kubitz and Pothakos, 1997), and such a biological state (i.e., increased resting EEG alpha power) is related to P3 potential (Bashore, 1989; Lardon and Polich, 1996; Polich, 1997). Taken together, these results indicate that acute exercise could change the serum cortisol concentrations and the level of alpha wave activity, which in turn modulate the P3 amplitude, as seen in the current study when the participants performed a cognitive task involving executive control after acute resistance exercise. However, the positive cortisol effect on P3 amplitude induced by acute exercise in the present study need to be explored in further experiments using longer regimes. In addition, although previous studies have not demonstrated the relationship between the P3 amplitude and cortisol levels, some studies demonstrated that ERN amplitudes predicted reduced cortisol increases during a Stroop task (Amen et al., 2008), while higher ERN amplitudes were associated with a greater decrease in cortisol during a task session (Tops et al., 2006). Therefore, the results of the current study might be able to explain how the reduced cortisol levels following acute resistance exercise could efficiently increase the P3 amplitude when the participants performed the cognitive task. However, it is interesting as such cortisol effects which seem to be specific to short burst exercise might have important ramifications for neurorehabilitation after brain injury and in the elderly.

Although the electrophysiological and biochemical findings of the present study could extend the current knowledge base regarding the beneficial effects of acute resistance exercise on behavioral performance, there are the following limitations to the current work which must be addressed. First, there are well-documented sex-specific differences in endocrine responses to acute resistance exercise, with higher basal GH levels and augmented (or at least equivalent) GH responses to a bout of acute resistance exercise found in males (Kraemer et al., 1991; Hakkinen et al., 2000). Further research is thus warranted in this area, possibly examining the relationships between acute resistance exercise and the neurotrophic factors in females. Second, since the blood samples in the current study were taken around 2–3 h after waking, the participants would have already experienced the large decrease in cortisol that occurs following the cortisol awakening response, which could lead to a favorable endocrine profile (Heaney et al., 2013). An identical experimental design, but carried out in the afternoon, is thus required to assess whether similar responses in cortisol levels after exercise would be found to those that the present study obtained in the morning. Third, since cortisol levels could also be influenced by an acute bout of aerobic exercise in young males (Kanaley et al., 2001), and such changes could affect cognitive performance (Kashihara et al., 2009), further research should explore the correlations between these in this context. In addition, since we only investigated the effects of acute exercise on the Go/No-Go task combined with the Erikson Flanker paradigm, it might also be worth considering using a broader array of cognitive measures and establishing whether the effects are different depending upon task demands.

In conclusion, this study found that a bout of moderate- or high-intensity resistance exercise could impact not only behavioral (i.e., RTs and accuracy rate) but also electrophysiological (i.e., P3 amplitude) performance in young male adults when performing a cognitive task involving executive functions. Although significantly different serum levels of neurotrophic factors (i.e., GH and IGF-1) could be secreted with different exercise intensities, there were no significant correlations between changes in the two neurotrophic factors and cognitive performance, possibly because the serum concentrations quickly returned to basal levels after exercise. The potential mechanisms underlying the changes in cognitive performance after acute resistance exercise found in the young male adults could be due to changes in arousal levels, possibly modulated by cortisol.

## Author contributions

Dr. Chia-Liang Tsai designed the study, wrote the protocol, and the first draft of the manuscript. Dr. Chun-Hao Wang analyzed the data. Dr. Chien-Yu Pan and Dr. Fu-Chen Chen worked on the revision of the manuscript. Dr. Tsang-Hai Huang helped collect and analyze the blood sample. Research assistant Mrs. Feng-Ying Chou helped collect data.

### Conflict of interest statement

The authors declare that the research was conducted in the absence of any commercial or financial relationships that could be construed as a potential conflict of interest.
